# Policy lessons from a vaping ban, Singapore

**DOI:** 10.2471/BLT.25.294852

**Published:** 2025-10-01

**Authors:** Shin Ling Wu

**Affiliations:** a School of Psychology, Faculty of Medical and Life Sciences, Sunway University, No. 5, Jalan Universiti, Bandar Sunway, 47500 Selangor Darul Ehsan, Malaysia.

Vaping has reshaped the global landscape of nicotine use. Marketed as safer than conventional cigarettes, electronic (e)-cigarettes have attracted millions of young people. The 2025 World Health Organization’s (WHO) report on the global tobacco epidemic cautions that youth use of e-cigarettes now surpasses adult use in many countries, with an estimated 87% of adolescents preferring flavoured vaping products.^1^ Social media marketing has accelerated this trend, normalizing vaping in youth culture. 

Despite more than 100 countries introducing some form of e-cigarette regulation, adolescent uptake continues to rise.^1^ This regulatory failure stems partly from mixed messaging. Positioning e-cigarettes as safer alternatives for adult smokers while warning against youth use creates a contradiction that weakens prevention campaigns. Only a minority of countries impose comprehensive restrictions on flavours, packaging and advertising, leaving powerful commercial drivers of youth demand largely intact. Without decisive action, governments risk cultivating a new generation addicted to nicotine.^1^

The government of Singapore has chosen a radically different path having banned vaping since 2018. In August 2025, the Prime Minister announced that vaping would be treated as a drug offence, with harsher penalties for possession, sale or use.^2^ This initiative reframes vaping not as a tobacco-control issue but as a matter of drug enforcement. By classifying e-cigarettes as controlled substances, the government removes the ambiguity that hampers other countries’ policies and sends a clear signal that vaping is unacceptable.

This approach carries both psychological and public health implications. Research on social norms shows that consistent, simple messaging is more effective at shaping behaviour than nuanced risk communication. Singapore’s strategy provides clarity where harm-reduction narratives often do not, and reflects an appreciation of addiction dynamics, as once nicotine dependence becomes established in adolescence, reversing it requires substantial intervention. 

Yet prohibition raises ethical and practical dilemmas. Harm-reduction advocates argue that adults should retain access to safer alternatives to cigarettes.^3^ At the same time, protecting adolescents is a public health imperative, especially given evidence that e-cigarettes expose young users to toxicants, disrupt brain development and heighten risks of addiction and neurological disorders.^4^ Singapore’s policy prioritizes youth protection over adult autonomy, a choice that will be closely scrutinized worldwide.

Prohibition has risks. Illegal markets may emerge, undermining enforcement and exposing young people to unregulated products. Former smokers who had switched to vaping may relapse to combustible tobacco. Adolescents caught vaping may face punitive consequences that stigmatize them rather than provide support. Yet prohibition also offers clarity, framing vaping not as a consumer choice but as a social harm requiring decisive control.

Singapore’s decision highlights three broader lessons. First, clarity of framing matters. By treating vaping as a drug issue, Singapore removes the messaging ambiguity. Consistent framing strengthens public health messaging and enforcement alike.

Second, prevention is easier than reversal. Decades of tobacco control demonstrate the difficulty of reversing entrenched patterns of use. Singapore’s approach reflects a belief in stopping the epidemic before it starts, a principle with global resonance.

Third, policies must balance trade-offs. Prohibition may protect adolescents, but it restricts adult smokers who might benefit from harm reduction. Each country must navigate this tension considering its cultural norms, legal frameworks and enforcement capacities. 

Singapore’s move should be rigorously studied. Researchers have a rare opportunity to examine how prohibition shapes adolescent behaviour and perceptions of risk, whether illicit trade emerges and how it is managed, and how adult smoking and cessation patterns are affected. Researchers will also be able to understand how citizens perceive the legitimacy of prohibition and how it shapes trust in public health authorities.

Such evidence will be invaluable for countries facing the same dilemma. Determining whether prohibition, regulation or hybrid approaches best serve public health requires careful monitoring, comparative evaluation and willingness to adapt policy in response to evidence. Singapore’s decision provides a test case whose outcomes will inform global practice.

Singapore’s criminalization of vaping marks a turning point in global nicotine policy. This radical approach reframes the debate of whether societies should accept some adult benefits of harm reduction at the cost of youth risk or draw a hard line against a new epidemic.

Whichever path other countries choose, Singapore’s experience will influence the next decade of global tobacco and drug policy. The country’s move creates a natural experiment whose outcomes will test whether prohibition can succeed where regulation has struggled. The responsibility now lies with researchers, policy-makers and citizens worldwide to document, analyse and debate its effects, ensuring that future decisions are guided by principle and evidence.
